# Imaging body composition in cancer patients: visceral obesity, sarcopenia and sarcopenic obesity may impact on clinical outcome

**DOI:** 10.1007/s13244-015-0414-0

**Published:** 2015-06-13

**Authors:** Connie Yip, Charlotte Dinkel, Abhishek Mahajan, Musib Siddique, Gary J. R. Cook, Vicky Goh

**Affiliations:** Division of Imaging Sciences & Biomedical Engineering, King’s College London, London, UK; Department of Radiation Oncology, National Cancer Centre, Singapore, Singapore; PET Imaging Centre, Guy’s & St Thomas’ NHS Foundation Trust, London, UK; Department of Radiology, Guy’s & St Thomas’ NHS Foundation Trust, London, UK; Department of Radiology, Imaging 2, Level 2, Lambeth Wing, St Thomas’ Hospital, London, SE1 7EH UK

**Keywords:** Sarcopenia, Sarcopenic obesity, CT, MRI, DXA

## Abstract

**Abstract:**

In recent years, there has been increasing interest in the influence of body composition on oncological patient outcomes. Visceral obesity, sarcopenia and sarcopenic obesity have been identified as adverse factors in cancer patients. Imaging quantification of body composition such as lean muscle mass and fat distribution is a potentially valuable tool. This review describes the following imaging techniques that may be used to assess body composition: dual-energy X-ray absorptiometry (DXA), computed tomography (CT) and magnetic resonance imaging (MRI). CT and MRI are acquired as part of oncological patient care, thus providing an opportunity to integrate body composition assessment into the standard clinical pathway and allowing supportive care to be commenced as appropriate to improve outcome.

***Main Messages*:**

• *Sarcopenia, sarcopenic obesity and visceral obesity are adverse prognostic factors in cancer patients.*

• *CT and MRI are the current gold standard in body composition evaluation.*

• *Body composition may affect chemotherapy tolerance and toxicities.*

## Introduction

Body composition is an important feature in cancer patients as it may affect the efficacy and toxicity of chemotherapy, and it is associated with patient outcomes in terms of functional status, surgical complication rates, length of hospital stay (LOS) and overall survival (OS) [1–8]. Assessment of body composition typically includes the quantitation of fat and muscle mass. In cancer patients, identification of risk factors including obesity (an increase in fat mass, in particular visceral fat mass), sarcopenia (loss of lean muscle mass and function) and sarcopenic obesity (a combination of loss of lean muscle mass and visceral obesity) will allow early supportive care such as dietary and/or physiotherapy interventions to be implemented.

### Obesity

The World Health Organisation body mass index (BMI) is most commonly used to define obesity [9]. The BMI is calculated by weight (in kilograms, kg) divided by the height (in metres, m) squared, where ≥40.0 kg/m^2^ equates to morbid obesity, 35.0–39.9 kg/m^2^ equates to class II obesity, 30.0–34.9 kg/m^2^ equates to class I obesity and 25.0–29.9 kg/m^2^ refers to overweight individuals [9]. However, associations between BMI and long-term outcomes and prognosis are weak in comparison to visceral obesity in cancer patients [5, 6, 10, 11]. In the non-oncological setting, the waist circumference (WC) and waist-to-hip ratio (WHR) have been found to be better discriminators of diabetes and cardiovascular risks compared to BMI [12, 13]. In the oncological setting, WC and WHR have been found to be associated with increased risk of endometrial, oesophagogastric, colorectal and breast cancers [14–16] although conflicting results were obtained in prostate and bladder cancers [14, 17]. There is a suggestion that WC and WHR are associated with inferior oncological outcomes such as colorectal cancer [18].

Visceral obesity refers more specifically to the excessive accumulation of visceral fat in the abdominal cavity [10, 11, 19, 20]. There is no definite normal range of visceral adipose tissue as this varies with age, gender, race and coexisting medical conditions [21]. Nonetheless, in one study that evaluated visceral adiposity on MRI in a predominantly Caucasian population, the 25th and 75th percentiles of intra-abdominal fat area were found to be 67.6–140.1 cm^2^ and 106.3–189.5 cm^2^ in females and males respectively [22]. Visceral obesity is calculated as the ratio of the visceral fat area to subcutaneous fat area, where a ratio greater than 0.4 is considered as visceral obesity [19]. Visceral fat differs from subcutaneous fat in that it has a higher number of large adipocytes, more glucocorticoid and androgen receptors, and is able to produce more free fatty acids in comparison to subcutaneous fat [19, 23, 24]. Visceral fat also secretes more bioactive molecules [13] and is associated with lower insulin sensitivity and higher circulating triglyceride levels [23–25] compared to subcutaneous fat. The link between visceral obesity and adverse outcomes in cancer patients may be partly due to increased insulin resistance and its influence on levels of endocrine hormonal secretion, which is also associated cancer progression [26, 27].

### Sarcopenia

Sarcopenia literally translates as ‘lack of’ (*penia*) ‘flesh’ (*sarco*) in Greek but refers to a loss of muscle mass as well as function. Sarcopenia may be primary (age-related) or secondary (associated with reduced activity, poor nutrition, malabsorption, endocrine disease, neurodegenerative disorders or cancer cachexia) [28–31]. Most frequently, sarcopenia has been defined as an appendicular skeletal muscle mass less than two standard deviations below the mean of a young healthy adult group as determined by dual-energy X-ray absorptiometry (DXA) [28, 30]. The European Society of Parenteral and Enteral Nutrition Special Interest Group (ESPEN SIG) proposed that sarcopenia should be diagnosed based on the presence of two criteria: (1) low muscle mass and (2) impaired muscular function [32]. Due to the significant variation in body composition between males and females, sex-specific skeletal muscle index cut-offs (52.4 cm^2^/m^2^ and 38.5 cm^2^/m^2^ for males and females, respectively) to define sarcopenia have been proposed in cancer patients and were shown to be associated with mortality [1]. It is worth bearing in mind that these definitions were derived from computed tomography (CT) images obtained at the level of the L3 lumbar vertebra. The use of these sex-specific cut-offs has been supported by an international consensus on the definition of cancer cachexia in 2011 [33]. It is important to be aware that sarcopenia may be present even in the absence of weight loss. For example, Prado et al. showed that 15 % of obese (defined as BMI ≥30 kg/m^2^) cancer patients were sarcopenic [1].

### Sarcopenic obesity

The combination of sarcopenia and obesity is classified as sarcopenic obesity. There are subtle variations in the exact definition of both conditions in various studies, depending on the method of assessment, although the latter is commonly defined as BMI ≥30 kg/m^2^ [34]. Several factors could increase the risk of sarcopenic obesity [34]. Age-related body composition changes with progressive decline in muscle mass and/or strength is a significant risk factor [34, 35]. Hormonal changes, sedentary lifestyle and malnutrition may also occur in the elderly, contributing to sarcopenic obesity. In addition, adipose tissue secretes pro-inflammatory cytokines and adipokines, promoting insulin resistance [34, 35], and these pro-inflammatory markers can contribute towards low muscle mass and obesity [36].

### Cachexia

Cachexia, derived from the Greek words ‘*cac*’ or bad and ‘*hexis*’ or condition, is well recognised in patients with chronic illnesses such as cancer, end-stage renal disease, cirrhosis and chronic obstructive pulmonary disease [30, 33]. The ESPEN SIG and Cachexia Consensus Working Group have defined cachexia as a complex metabolic syndrome in chronically ill patients, associated with loss of muscle mass with or without loss of fatty tissue [32, 37]. Many proposed factors are involved in the development of cachexia such as chronic inflammation, increased muscle protein breakdown and insulin resistance [33, 37]. Although it may be difficult to differentiate sarcopenia from cachexia particularly in the oncological setting, most cachectic individuals have sarcopenia but not all sarcopenic patients are cachectic [30, 32].

## Assessment of body composition in clinical practice

Various techniques may be used to estimate body composition. These include bioimpedance analysis (BIA), DXA, CT and magnetic resonance imaging (MRI). CT and MRI are currently considered the gold standards for estimating muscle mass [30]. Both imaging modalities are obtained as part of the standard patient care pathway from tumour staging to response assessment and surveillance, thus providing an excellent opportunity to integrate body composition assessment into current patient care. The merits and disadvantages of the various techniques are summarised in Table 1.

**Table 1:** Summary of the various techniques used in body composition analysis

Techniques	Advantages	Disadvantages
**Bioelectrical impedance analysis (BIA)**	Inexpensive Portable Less time consuming No radiation exposure Immediate results	Lack of precision Skeletal muscle quality cannot be analysed
**Dual-energy X-ray absorptiometry (DXA)**	Inexpensive Low radiation exposure (equivalent to 3 days background radiation) More sensitive than BIA	Lack of portability Two-dimensional data Low precision compared to CT and MRI Distinction between subcutaneous and visceral adipose tissue cannot be made Skeletal muscle quality cannot be analysed
**Computed tomography (CT)**	High accuracy and reproducible results Lean body mass, subcutaneous fat and visceral fat can be defined	Radiation exposure More expensive compared to BIA & DXA Skeletal muscle quality cannot be assessed
**Magnetic resonance imaging (MRI)**	Best spatial resolution and body mass composition differentiation No radiation exposure	More expensive compared to BIA and DXA Longer image acquisition time Contraindications to MRI may preclude some patients Skeletal muscle quality cannot be analysed

### Anthropometrics and bioimpedance analysis

Anthropometric methods such as skin fold thickness by caliper measurement, mid arm and calf circumferences have been used to assess muscle mass. However, these methods are prone to measurement error with significant interobserver variability and are not recommended for routine diagnosis of sarcopenia [30].

BIA may be used to estimate fat mass relative to lean body mass [30]. This involves placing electrodes on the skin, e.g. of the hand and foot, and measuring the impedance of an applied low level electric current. The impedance is higher for fat and bone compared with soft tissue [30]. Impedance measurement can be affected by hydration status [38] and thus BIA should be performed under standard conditions to minimise the measurement variation.

A Japanese group has proposed sex-specific equations to estimate the appendicular skeletal mass using BIA: 0.197 × (impedance index) + 0.179 × (weight) – 0.019 (males) and 0.221 × (impedance index) + 0.117 × (weight) + 0.881 (females) [39]. However, it should be noted that these equations were derived from the older Japanese population and have not been validated in other populations or in the oncological setting. The same group also defined the skeletal muscle mass index as appendicular skeletal mass/height^2^ [40]. They classified those with a skeletal muscle mass index less than 7.09 kg/m^2^ in males and 5.91 kg/m^2^ in females as sarcopenic based on the lowest sex-specific 20 % quintiles in the healthy population [40].

### Dual-energy X-ray absorptiometry

**Fig. 1:**
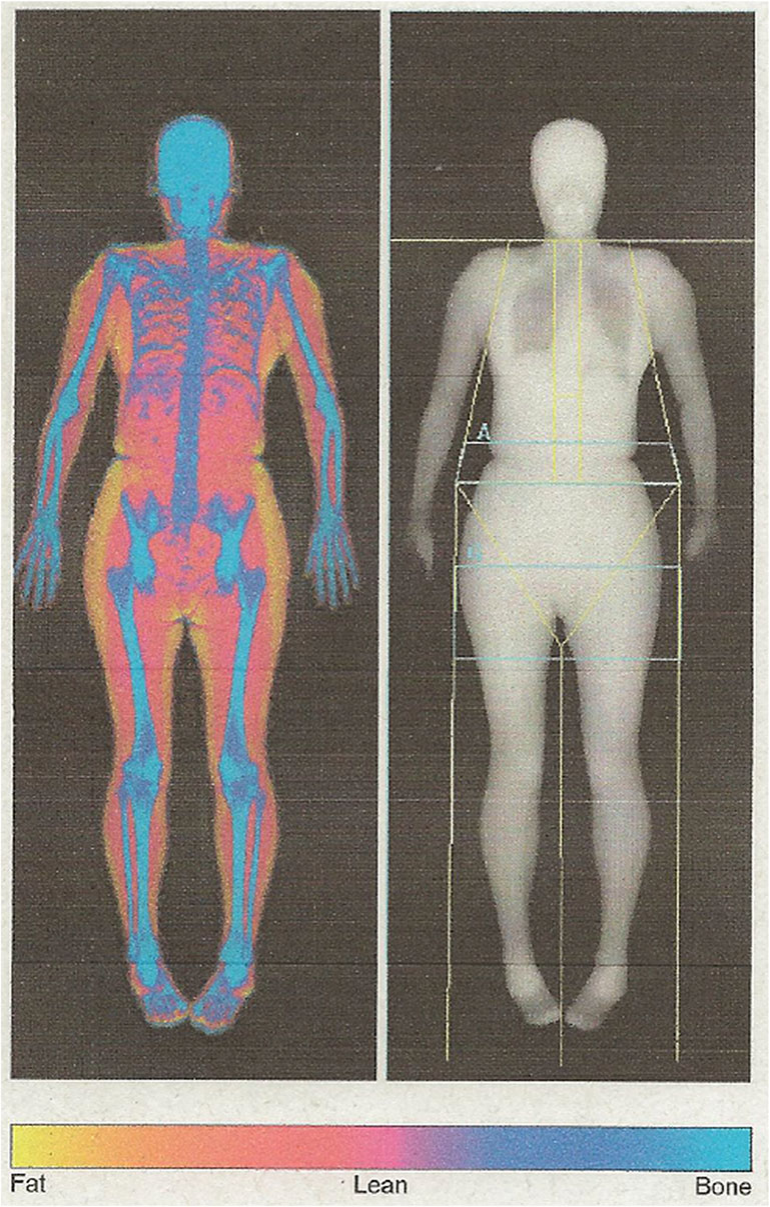
Whole-body DXA image showing lean, fat and bone masses

Dual energy X-ray absorptiometry (DXA) exploits the difference in the attenuation of tissue and bone at different X-ray energies to measure lean body mass (LBM), fat mass (FM) and bone mineral mass (BMM) [41], which can be extrapolated to the whole body (Fig. 1). An X-ray source produces a fan beam at two average X-ray energies, typically 30–40 keV and 70–90 keV, depending on whether filtration or 70 kV/140 kV tube voltage switching is used [42]. The typical radiation exposure of a DXA scan is low (0.1 mSv). The X-ray attenuation and transmission reflect the differences in tissue thickness, density and the elemental composition of the different compartments. Attenuation increases with tissue thickness and is greater for bone than soft tissue.

DXA is widely used as a clinical tool but is associated with some limitations. The DXA scan produces a two-dimensional image; therefore distinction between subcutaneous and visceral adipose tissue cannot be made. Certain assumptions have to be made, such as the extent of distribution of the fat and muscle compartments, particularly where there is overlying bone. As with any quantitative imaging technique, different manufacturers' software, calibration methods, calculation algorithms and scanners may result in variation in the calculated estimates of body composition [41, 43–46].

### Computed tomography and magnetic resonance imaging

**Fig. 2:**
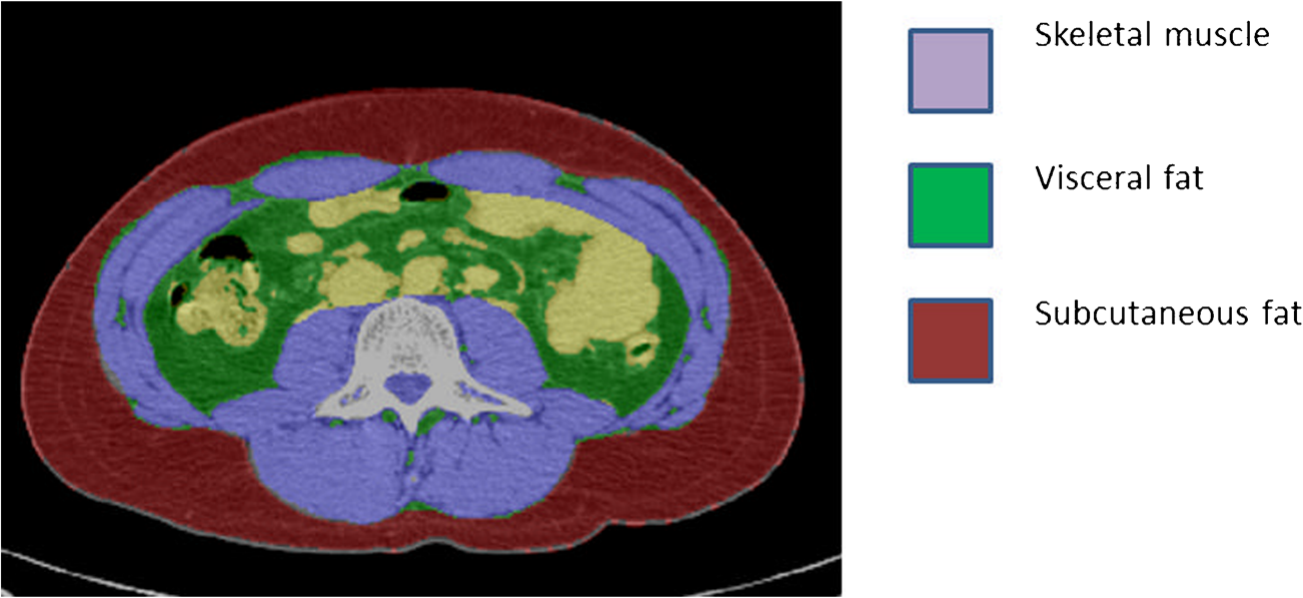
Subcutaneous fat, visceral fat and skeletal muscle as depicted on an axial CT image at the level of L3 vertebral body

CT and MRI are high spatial and contrast resolution cross-sectional techniques that can provide estimates of lean muscle mass and adipose tissue as well as fat infiltration within the skeletal muscle [47–50]. The methods used in the measurement of cross-sectional body composition are similar for CT and MRI. The user is usually required to manually delineate the fat or muscle compartment of interest on a dedicated software platform (Fig. 2). These regions of interest are then further refined using specific Hounsfield unit (HU) segmentation thresholds in CT [1] or grey-level value thresholds in MRI, the latter requiring more complicated segmentation algorithms [51, 52].

#### CT

The performance of CT in body composition analysis has been shown to be superior to DXA [49]. Lean body mass, subcutaneous and visceral fat mass can be delineated for a given volume on CT images (Fig. 2) and extrapolated to the whole body [49]. Several parameters such as the total fat mass, total lean body mass, fat and lean body mass indices (normalised for stature), subcutaneous fat-to-muscle ratio and visceral-to-subcutaneous adipose tissue ratio may also be derived.

Total body fat mass and lean body mass may be defined using the following equations [49]:

$$ \begin{array}{l}\mathrm{Total}\ \mathrm{body}\ \mathrm{fat}\ \mathrm{mass}\ \left(\mathrm{kg}\right) = 0.042 \times \left[\mathrm{total}\ \mathrm{adipose}\ \mathrm{tissue}\ \mathrm{at}\ \mathrm{L}3\ \left({\mathrm{cm}}^2\right)\right] + 11.2\hfill \\ {}\mathrm{Total}\ \mathrm{body}\ \mathrm{lean}\ \mathrm{body}\ \mathrm{mass}\ \left(\mathrm{kg}\right) = 0.3 \times \left[\mathrm{skeletal}\ \mathrm{muscle}\ \mathrm{at}\ \mathrm{L}3\ \left({\mathrm{cm}}^2\right)\right] + 6.06\hfill \end{array} $$

The L3 lumbar vertebra landmark is often used in cross-sectional body composition analysis and is found to correspond to the whole-body tissue measurements [50, 53]. The field of view at this vertebral level includes the psoas, paraspinal muscles (erector spinae, quadratus lumborum) and abdominal wall muscles (transversus abdominus, external and internal obliques, rectus abdominus), thus making it an optimal level for skeletal muscle quantification. However, while the L3 vertebral level is also used to assess fat mass, the amount of fat will vary according to sex, age and body level. Thus, there are suggestions that visceral fat should be derived by obtaining measurements at several different anatomic levels [54] although others have found no significant clinical impact when correlating visceral fat measured at L2-L3, L4-L5 and mid waist levels and patient outcome [55].

There is no defined guideline on the image acquisition parameters that are required for the purpose of cross-sectional body composition analysis. Thus, for patients who are undergoing abdominopelvic CT as part their routine diagnostic or management algorithm, the following standard CT acquisition parameters are appropriate: 120 kV, variable mA with dose modulation, soft tissue reconstruction algorithm, matrix of 512 × 512, field of view (FOV) of 30-35 cm and reconstructed slice thickness 5 mm. However, for patients undergoing a targeted CT solely for the assessment of body composition, a limited low-dose axial 10-mm acquisition at the L3 level may be appropriate: 120 kV, <80 mAs, soft tissue reconstruction algorithm, matrix of 512 × 512 and FOV of 30-35 cm with the advantage that the additional radiation exposure from a limited CT is small and is equivalent to a chest radiograph [56].

#### MRI

The major advantage of MRI over CT in body composition analysis is its lack of radiation exposure. However, the use of MRI is limited by the local availability and technical expertise. Nonetheless, clinical MRI scanners are more widely available now and whole-body MRI techniques are being introduced that could represent a step forward in MRI assessment of body composition. MRI has better soft tissue definition particularly of adipose tissue compared to CT as fat has short T1 and long T2 proton relaxation times [57] and thus may improve image segmentation of adipose tissues and skeletal muscle. The majority of the published literature on the use of MRI has evaluated its use in fat mass analysis [52, 58, 59]. MRI estimation of subcutaneous and intra-abdominal adipose tissues has been shown to correlate with direct measurement of the corresponding cadaveric tissues [60]. Similar to CT, MRI evaluation at the level of the L2/3 vertebra was found to be a reliable estimate of fat mass [61].

**Fig. 3:**
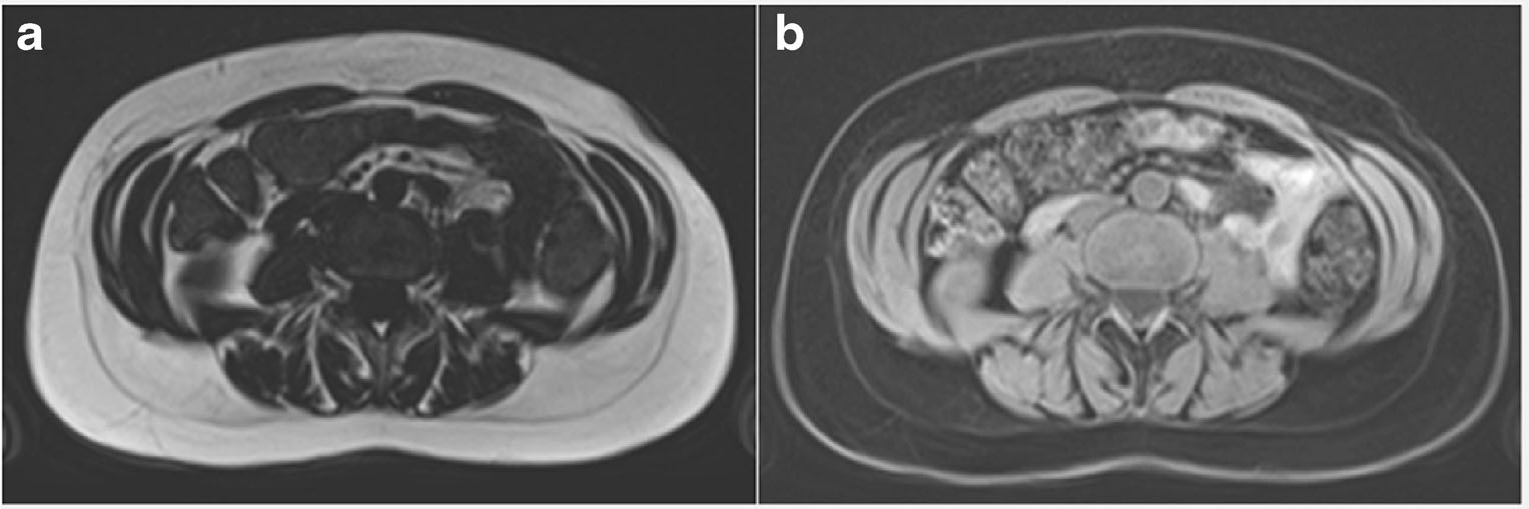
T1-weighted axial DIXON MRI images highlighting (**a**) fat and (**b**) water signals at the level of the L3 vertebral body

Improved segmentation of MRI fat and lean body mass may be produced using a two-point DIXON method for fat/water separation (Fig. 3) [62, 63]. The information from in- and out-of-phase gradient echo sequences may be combined. In the in-phase image, the signal (S_ip_) represents the sum of fat (S_f_) and water (S_w_) signals, i.e. S_ip_ = S_w_ + S_f_, while the out-of-phase signal represents the difference, i.e. S_ip_ = S_w_-S_f_. Averaging of the sum and difference of the in- and out-of-phase images will result in water and fat signal respectively. A correction for T2* differences is required as the two images have different echo times. The two-point DIXON method assumes that the main field B_0_ homogeneity is perfect. However, this is not true and refinements such as a three-point method have been proposed where an in-phase TE sequence is used to correct for B_0_ field homogeneity [64].

A simpler and quicker way of assessing whole-body fat mass and possibly lean body mass may be feasible with the advent of whole-body MRI and newer computer-aided methods of image segmentation [52]. This may have potential clinical utility as whole-body MRI is currently being evaluated as a staging modality in different tumour sites such as multiple myeloma.

A general limitation of these imaging techniques is that they only provide anatomical information and not functional information such as muscle function. Thus, these imaging findings have to be considered in conjunction with formal assessment of muscle function, particularly in the diagnosis of sarcopenia [32]. However, there is a suggestion that skeletal muscle attenuation on CT (Hounsfield units, HU) may potentially be a surrogate for muscle function [65, 66], with reduced HU within skeletal muscle representing increased intramuscular lipid deposition, which has been observed in those with neuromuscular disease [65]. This is still an area of research and no definite HU cut-offs have been reliably identified to represent reduced muscle function for this to be adopted in the clinical setting at present. Reduced skeletal muscle attenuation has also been found to be a negative prognostic factor in patients with gastrointestinal and respiratory tract cancers [67].

## Clinical applications in oncology

### Body composition as a prognosis marker

To our knowledge, there is no published study looking at the use of MRI body composition assessment in cancer patients. CT is the most commonly used cross-sectional imaging technique in this setting and we will be focusing on its use in this section.

Sarcopenic cancer patients have been shown to have higher rates of morbidity and mortality [1, 4, 6, 8, 10, 48]. Lieffers et al. showed that patients with colorectal cancer were at risk of adverse outcomes after primary colorectal surgery if they had co-existing sarcopenia [4]. In this study, more than a third of the patients (39 %) were found to be sarcopenic and they had an increased length of stay in hospital (mean 16 ± 14 days) compared to the non-sarcopenic patients (12 ± 10 days, *p* = 0.038). Post-operative infection risk was also higher in those with sarcopenia (24 % vs. 13 %, *p* = 0.025). These risks were more pronounced in patients aged 65 years and above. However, visceral and subcutaneous adiposities were not significant predictors of length of stay and postoperative complications in a separate study [5].

Moon et al. showed that the visceral fat area-to-subcutaneous fat area ratio was a significant prognostic factor in predicting disease-free survival in patients with resectable colorectal cancer [6]. Those with visceral fat area-to-subcutaneous fat area ratio >0.5 had shorter disease-free survival (HR 1.98, 95 % CI 1.02-3.87, *p* = 0.044) although it did not have a significant impact on overall survival. Similarly, viscerally obese patients (visceral fat area-to-subcutaneous fat area ratio ≥0.4) with rectal cancer had poorer disease-free survival (HR 3.50, 95 % CI 1.12 – 10.17, *p* = 0.09) but there was no significant difference in overall survival [10]. In contrast, body mass index measurements did not correlate with any survival outcomes [6, 10].

Prado et al. found that sarcopenic obesity was a significant prognostic factor in patients with gastrointestinal and respiratory tract cancers [1]. Patients with coexisting sarcopenic obesity had poorer functional status (*p* = 0.009) and overall survival (HR 4.2, 95 % CI 2.4–7.2, *p* < 0.0001). These findings were confirmed in patients with advanced pancreatic cancer [48]. In this study, sarcopenic obesity was a significant predictor of reduced overall survival (HR 2.07, 95 % CI = 1.23–3.50, *p* = 0.006).

In contrast to the above positive findings, fat mass and fat-free mass were not associated with in-hospital mortality or survival in patients with gastro-oesophageal (GOJ) cancer treated with neoadjuvant chemotherapy [68]. However, it is noteworthy that the authors evaluated the prognostic value of pre-treatment and post-treatment fat-free mass only. Whether fat mass, as shown in the colorectal cancer population, could have a greater prognostic impact in GOJ cancer remains unclear. In addition, it may be that a reduction in fat-free mass during treatment may be a more important prognostic factor than the absolute baseline or post-treatment values as evaluated in this study.

### Treatment implications

**Fig. 4:**
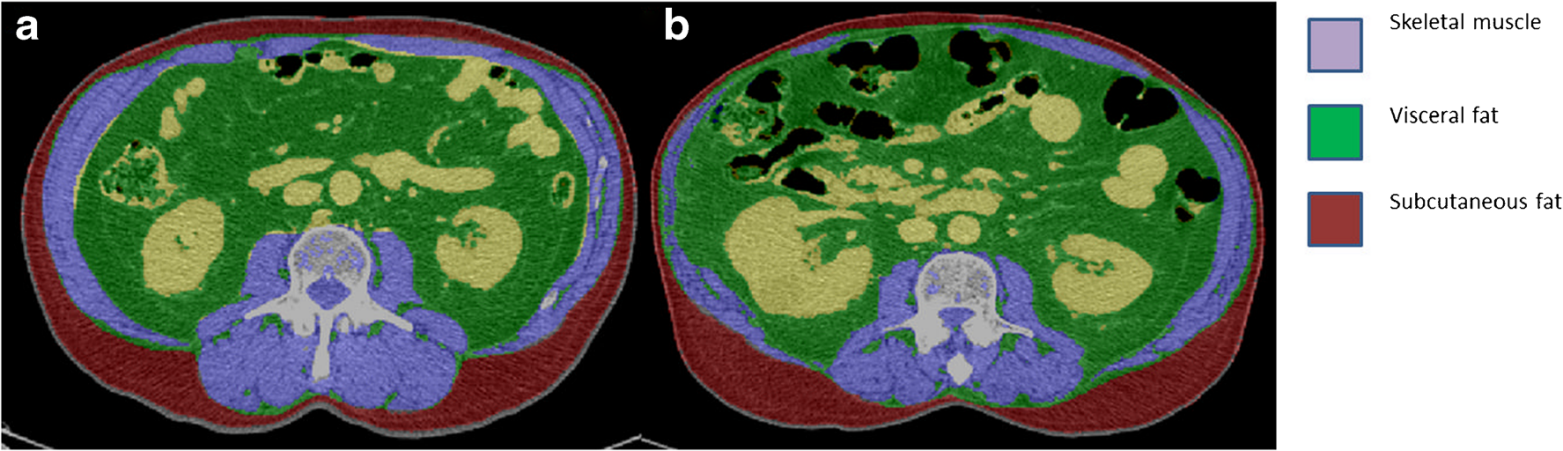
Axial CT images at the level of L3 vertebra demonstrating progressive sarcopenia in a patient with oesophageal cancer before (**a**) and after (**b**) neoadjuvant chemotherapy. There is a loss of abdominal muscle mass with an increase in visceral fat

In addition to its potential prognostic impact, body composition may also affect an individual’s tolerance to non-surgical treatment and could be predictive of treatment toxicity. First, the use of chemotherapy has been shown to alter body composition [69]. Yip et al. showed that fat mass and fat-free mass, as measured using CT, and weight decreased after neoadjuvant chemotherapy in patients with oesophageal cancer (Fig. 4). Similarly, a separate study demonstrated that fat mass and fat-free mass decreased but the proportion of patients with sarcopenic obesity increased following neoadjuvant chemotherapy in those with GOJ cancers [68].

These body composition changes could have an important impact on patient’s tolerance to subsequent therapy as sarcopenia may increase the risk of chemotherapy toxicity [2, 3, 70]. At present, the body surface area is used to calculate cytotoxic chemotherapy dosing. As with the body mass index, the body surface area is derived using the patient’s height and weight but is associated with many limitations, particularly in those with extreme variation in body composition such as obese patients, leading to under- or overdosing [71]. The risk of toxicity is higher in female patients as they tend to have a lower lean body mass in comparison to their body surface area [2]. This has led to recent suggestions that lean body mass may be a better measure to dose chemotherapy on an individual patient basis [2, 70].

Prado et al. showed that lean body mass was a significant predictor of dose-limiting toxicities in patients treated with 5-fluorouracil (5FU) and leucovorin for stage II/III colon cancer [2]. In this study, females who had more than 20 mg of 5FU/kg lean body mass were found to have lower cross-sectional muscle mass and LBM on CT. The authors found that a 5FU/kg lean body mass cut-off value of less than 20 mg/kg was a significant predictor of toxicity. This is possibly due to the differential drug distribution in the different body compartments as hydrophilic drugs are distributed into the lean body compartment whereas lipophilic drugs are distributed into the fat compartment. Thus, the size of these compartments, which could be easily assessed using CT, would affect drug distribution and therefore toxicity.

The same group also evaluated the impact of sarcopenia on toxicity and time to progression in patients with metastatic breast cancer treated with capecitabine [70]. They found that 50 % of sarcopenic patients had grade 2 or greater toxicities compared to 20 % of non-sarcopenic patients (*p* = 0.03). Sarcopenic patients also had significantly shorter time to progression compared to the non-sarcopenic cohort (median 62 days vs. 105 days, *p* = 0.05). Similar observations were also noted in patients treated with sorafenib for metastatic renal cell carcinoma [3]. In this study, a greater proportion of sarcopenic male patients experienced dose-limiting toxicities during sorafenib therapy compared to the non-sarcopenic patients (37 % vs. 5 %, *p* < 0.04).

An objective assessment of body composition using cross-sectional imaging techniques such as CT and MRI has the potential to complement our current clinical and nutritional evaluation of patients’ fitness and treatment tolerability. This information can be readily obtained from standard diagnostic scans performed during the various stages of patient care. Nutritional support can then be initiated at an earlier and appropriate stage, which could improve treatment compliance and clinical outcome.

### Other metabolic associations

Although not a direct oncological implication, the metabolic effect of body composition on cardiovascular risk and mortality will have an impact on the patients’ overall life expectancy and tolerance to oncological treatment. Anthropometric indices such as BMI, WC and WHR are associated with cardiovascular risk factors such as hypertension, diabetes and dyslipidemia, and cardiovascular disease [12, 72]. Similarly, visceral adipose tissue as defined on CT has been shown to be associated with adverse cardiovascular risk factors [25]. These associations should be considered in order to provide a holistic approach to patient care.

## Future directions

There is sufficient evidence to support the use of body composition assessment to direct and improve supportive oncological care such as dietary intervention and physiotherapy support. As cross-sectional body composition evaluation is straight forward, this can be introduced with relative ease into a routine oncology report particularly in high-risk patients with gastrointestinal cancers or pre-existing gastrointestinal disease. However, the use of body composition in modifying cancer therapy requires further research preferably as prospective clinical studies.

## Conclusions

In conclusion, sarcopenia, sarcopenic obesity and visceral obesity may be associated with negative oncological outcomes. Imaging assessment of body composition can be readily applied in the clinical setting with the potential to improve individual nutritional care and perhaps chemotherapy dose calculation. This personalised cancer management strategy may reduce treatment-related toxicities and ultimately improve patient outcomes.
